# Effect of Silica Fume in Concrete on Mechanical Properties and Dynamic Behaviors under Impact Loading

**DOI:** 10.3390/ma12193263

**Published:** 2019-10-07

**Authors:** Shijun Zhao, Qing Zhang

**Affiliations:** 1 College of Mechanics and Materials, Hohai University, Nanjing 211100, China; zhaoshijun@hhu.edu.cn; 2 Department of Mechanical and Materials Engineering, University of Nebraska-Lincoln, Lincoln, NE 68588, USA

**Keywords:** concrete, silica fume, mechanical properties, dynamic behaviors, impact loading, split Hopkinson pressure bar test

## Abstract

The effect of silica fume (SF) in concrete on mechanical properties and dynamic behaviors was experimentally studied by split Hopkinson pressure bar (SHPB) device with pulse shaping technique. Three series of concrete with 0, 12%, and 16% SF as a cement replacement by weight were produced firstly. Then the experimental procedure for dynamic tests of concrete specimens with SF under a high loading rate was presented. Considering the mechanical performance and behaviors of the concrete mixtures, those tests were conducted under five different impact velocities. The experimental results clearly show concrete with different levels of SF is a strain-rate sensitive material. The tensile strength under impact loading of the tested specimens was generally improved with the increasing content of SF levels in concrete. Additionally, the tensile strength under impact loading of the concrete enhances with the increase of the strain rates. Finally, failure modes, dynamic tensile strength, dynamic increase factor (DIF), and critical strain are discussed and analyzed. These investigations are useful to improve the understanding of the effect of SF in concrete and guide the design of concrete structures.

## 1. Introduction

Concrete is currently the most commonly used construction and building material. Dynamic resistance is a fundamental basis for the evaluation of structural safety. The responses of concrete to transient dynamic loading (including compressive and tensile loading) are of interest in both academic and engineering fields, such as bridge construction, hydraulic engineering, constructional engineering, and so on [1,2,3]. To better understand the mechanical properties and dynamic behaviors of concrete under dynamic tensile loading is a greatly significant requirement in civil and military protection engineering. Using natural or artificial pozzolans in combination with ordinary Portland cement (OPC) to obtain high-performance concrete is an effective way, which mainly aims to develop the mechanical properties of concrete, such as strength, permeability, sustainability, and durability [4,5,6,7]. Therefore, it is meaningful to understand the use of silica fume (SF) and other auxiliary cementitious materials. SF is a kind of material that can improve the durability, mechanical properties, and behaviors of concrete [8,9]. The average particle size of SF is relatively small, with good filling effect and can be filled between the cement particle gaps. At the same time, the production of gel, water, and alkaline materials, including magnesium oxide, can enhance the strength and durability of concrete. The amount of SF in concrete and mortar can significantly improve its compressive strength, flexural, anti-permeability, corrosion resistance, and abrasion [10,11]. Moreover, SF is comprised of amorphous spherical particles which enhance the rheological properties of concrete. Considering the above characteristics, SF is a highly reactive pozzolanic material and has been studied as a partial substitute for cement in concrete. Adding SF into the concrete mixture can reduce the porosity, permeability, and bleeding rate of concrete [12,13]. 

Due to the different mixing methods and amounts, the influence of SF on the mechanical properties and behaviors of concrete is quite different. Recent investigations have tried to improve the mechanical properties and behaviors of concrete by using SF for cement replacement. Pedro et al. [14] investigated and evaluated the effect of SF on the behaviors of high-performance concrete. They found that the mechanical properties of concrete were improved when SF replaced cement. Shannag [9] found that the certain natural pozzolan-silica fume combinations can improve the compressive and splitting tensile strengths, workability, and elastic modulus of concrete. Ramezanianpour [15] studied the effect of combined carbonation and chloride ion ingress by an accelerated test method on the microscopic and mechanical properties of concrete. According to Bingol and Tohumcu [16], increasing the replacement percentage of SF in concrete can result in increased compressive strength. SF has positive effect on self-settlement properties. Ghahari [17] investigated the performance of roller compacted concrete (RCC) containing Trass, as a supplementary cementitious material, and an air-entraining agent for salt scaling. Okoye et al. [18] found that a geopolymer concrete with SF presented higher compressive strength, tensile strength, and flexural strength. These strength values increased with the increasing addition of SF levels. An experimental study carried out by Sarıdemir [10] indicated that high-strength concrete can be obtained with SF and SF together with ground pumice content. More emphasis has focused on static or quasi-static loading. However, there are few investigations reporting on the mechanical properties and dynamic behaviors of concrete with SF under high strain rates. In addition, the effect of SF is the most qualitative description from previous investigations. In this context, we distinguished the effect of the content of SF in concrete by quantitative description, which is meaningful to understand the influence and effects of SF in concrete under impact loading.

Split Hopkinson pressure bar (SHPB) device is an effective technique to analyze and characterize the mechanical properties and dynamic behaviors of brittle materials at high strain rate. In recent years, researchers studied the dynamic mechanical properties of brittle materials, such as rock or rock-like materials [19,20,21,22,23], concrete-like materials [24,25,26,27,28], and ceramics materials [29,30], by using an SHPB device under strain rates ranging from 10^2^ to 10^4^ s^−1^. Many factors have obvious influences on the strain rate sensitivity of concrete. The strain rate sensitivities are mainly measured by strength or the strains at the maximum stress [31,32,33,34,35,36,37], and the dynamic compressive strength and impact toughness increase with the strain rate. 

This study uses Brazilian disk (BD) specimens containing different levels of SF, using an SHPB test device, and proposes to enhance the understanding of SF in concrete on the mechanical properties under impact loading. At the same time, it intends to promote and evaluate the use of SF to replace cement in concrete. For those reasons, three series of concrete mixtures with different SF levels were produced and tested. The materials’ strains and derived testing strain rates were recorded by resistance strain gauges which were placed on the surface of elastic bars. The influences of the strain rates on the mechanical properties and dynamic behaviors of the tested specimens with different mixture proportions of SF were studied. This present study is organized as follows: Section 2 is the tested concrete specimens’ preparation and experimental process. In Section 3 we present the testing results (failure patterns of tested specimens, stress-strain curves, strain rate, DIF, and critical strain). The conclusions obtained from this experimental investigation are presented in Section 4.

## 2. Materials and Methods 

### 2.1. Materials

During this experimental study, five kinds of materials were consumed: cement, fine aggregates, coarse aggregates, water, and SF. In this study, the cement is ordinary Portland cement (OPC), supplied by Anhui Conch Cement Company, Wuhu City, Anhui Province, China. The specific density of the OPC is 3.15 g/cm^3^. The supplementary cementitious materials for concrete are OPC and SF. The particle size of fine aggregates ranges from 0.5 mm to 2.5 mm with continuous gradation, of which the density is 2.64 g/cm^3^. The water is potable water. Coarse aggregates are natural crushed stones having rough surfaces and angular shapes. The maximum particle size of coarse aggregates is 10 mm, of which the density is 2.65 g/cm^3^. In order to study the influences of SF on the mechanical properties of concrete, the concrete was distributed into I, II, and III series. Details of the mix proportions of the three series of concrete are listed in Table 1. Chemical compositions, and the physical properties of OPC and SF are given in Table 2.

### 2.2. Curing of the Specimens

The concrete was produced and cured in accordance with BS EN 12390-2 [38] and EN 12390-6 [39]. Aggregates and other binders were mixed together by mixing steel pans in dry conditions. For this work, the concrete was poured in the cuboid-shaped steel molds with a size of 200 mm × 100 mm × 100 mm. After production, all the cuboid-shaped concrete was cured in water tanks under standard laboratory condition (the temperature was 20 ± 2 °C and 70% relative humidity). Based on recommendations from the Concrete Society, concrete specimens with SF need to be moist cured for no less than seven days. After being de-molded at the age of 72 h, all the cuboid-shaped concrete was cured in tanks with water until the age of 28 days at laboratory temperature.

### 2.3. Production of the Specimens

**Figure 1:**
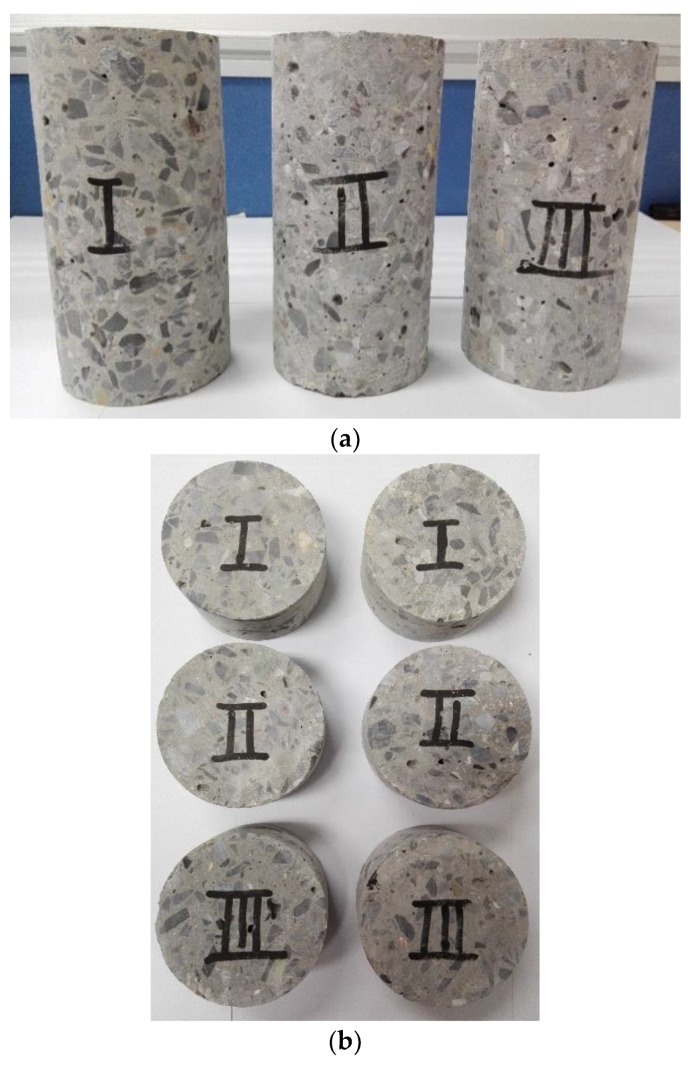
Images of the series I, II, III concrete BD specimens: (**a**) For quasi-static loading tests; (**b**) For impact loading tests.

The geometries of the concrete specimens are *Φ* 75 mm × 37 mm for dynamic loading tests, and *Φ* 75 mm × 150 mm for quasi-static loading tests. Firstly, the cuboid-shaped concrete was cored into a cylindrical shape with a diameter of 75 mm and then cut into a Brazilian disk (BD) shape with a thickness of 37 mm. The BD specimens’ ends were ground to achieve the parallelism of the specimen surface. To determine the effect of SF in concrete, significant effort was made to distinguish the BD specimens with different SF content. Three specimens for each test were prepared to guarantee the reproducibility and reduce the discreteness of those experimental results. A total of 45 specimens (series I: 15 specimens, series II: 15 specimens, series III: 15 specimens) were prepared for high strain rates impact loading tests. Another 18 specimens (series I: 6 specimens, series II: 6 specimens, series III: 6 specimens) were prepared for quasi-static loading tests. Images of the concrete BD specimens is shown in Figure 1. 

The operating principle of the SHPB equipment is based on one-dimensional wave transmission theory, which is satisfied with two hypotheses: the stress and strain propagate uniformly along the axis, and the inertia and friction effect of the specimen can be ignored. It is necessary to reduce the diameter of specimens to obey the rationality of the above assumptions. In the SHPB test, the size of the specimens is usually 75 mm with little fluctuation. Moreover, the concrete specimens usually contain aggregate particles. To ensure the reasonableness and accuracy of the mechanical properties, the minimum diameter of the specimens must not be less than three times the maximum aggregate size. In addition, from the perspective of reducing the inertia effect of the specimens, the length of the specimen should be as small as possible.

### 2.4. Test Method

Quasi-static loading tests were conducted by an MTS testing machine (manufactured by MTS System Corporation, Eden Prairie, MN USA). Table 3 shows the tested results (the values are averaged ones). Due to the high-strength concrete being more difficult to destroy than low-strength concrete under low impact velocities, the impact velocities we employed are low in the tests. The computer system of the MTS testing machine controls the rotation of the servo motor through the controller and the speed-regulating system, and drives the moving beam to rise and fall through the precision screw pair after the deceleration system, completing the tensile, compression, bending, shearing, and other mechanical properties tests of the sample. The technical parameters and accuracy of the MTS testing machine are listed in Table 4.

**Figure 2:**
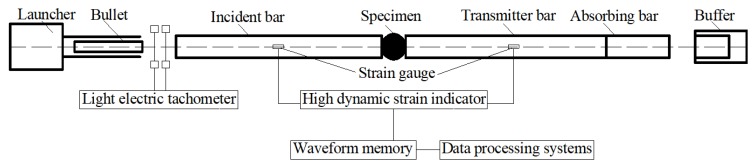
Schematics of the 74 mm-diameter SHPB test device.

Dynamic loading tests under high strain rates were carried out by the SHPB test system. The experimental procedure was conducted by a *Φ* 74 mm-diameter straight taper variable cross-section SHPB device at Hohai University, Nanjing, China. The SHPB test device is comprised of the following parts: three elastic bars (including an incident bar, a transmitter bar, and an absorbing bar), power systems (including an air compressor and pressure vessel) which is propelled by a gas gun, buffer (energy-absorbing device), and data processing systems (including strain gauges, a high-dynamic strain indicator, and wave-form memory). The bullet’s velocities (equal to the impact velocity) can be captured by light-electric tachometers. The technical parameters and accuracy of the SHPB testing system is listed in Table 5. During tests, the resistance strain gauges were placed on the surface of the elastic bars to collect the specimens’ strains [6]. Schematics of the *Φ* 74 mm-diameter SHPB system are presented in Figure 2. In SHPB tests, the stress $\sigma_{s}\left(t\right)$, strain $\varepsilon_{s}\left(t\right)$, and strain rate $\dot{\varepsilon }\left(t\right)$ of the specimens can be calculated by the following equations [40]:

(1) $$\sigma_{s}\left(t\right)=\frac{S_{B}E}{{2S}_{S}}\left[\varepsilon_{t}\left(t\right)+\varepsilon_{r}\left(t\right)+\varepsilon_{i}\left(t\right)\right]$$

(2) $${\dot{\varepsilon }}_{t}\left(t\right)=\frac{C_{0}}{L_{s}}\left[\varepsilon_{t}\left(t\right)+\varepsilon_{r}\left(t\right)-\varepsilon_{i}\left(t\right)\right]$$

(3) $$\varepsilon_{t}\left(t\right)=\frac{C_{0}}{L_{s}}\int_{0}^{t}\left[\varepsilon_{t}\left(t\right)+\varepsilon_{r}\left(t\right)-\varepsilon_{i}\left(t\right)\right]d\tau$$

where $S_{B}$, E, $C_{0}$ are the elastic bars’ cross-sectional area (mm^2^), Young’s modulus (GPa), and elastic wave velocities (km/s); $L_{s}$, $S_{s}$ are the concrete specimens’ length (mm) and cross-sectional area (mm^2^); $\varepsilon_{i}\left(t\right)$, $\varepsilon_{r}\left(t\right)$, $\varepsilon_{t}\left(t\right)$ are the captured strains of the tested concrete specimens.

Before stress is uniformly reached, tested specimens can be fractured in SHPB tests. Therefore, modification of the incident pulse technique is required to match the elastic response. In this work, the pulse-shaping technique (a thin copper disk, with a size of 12 mm diameter and 1 mm thickness) was applied in the SHPB tests. The pulse-shaping copper disks can improve the stress wave shapes through attenuating high-frequency oscillations of the incident stress waves [18,19]. The pulse-shaping copper disks reduce the pulse distortion in the elastic bars and smooth the waveforms. As a result, the tested specimens can reach stress uniformity before fracturing [41]. The thin copper disk glued on the incident bar can extend the rising time of the incident wave, reduce the loading rate, and capture uniform stress and strain in the tested specimens. The principles and functions of the pulse-shaping technique have been discussed in detail by Chen et al. [26].

**Figure 3:**
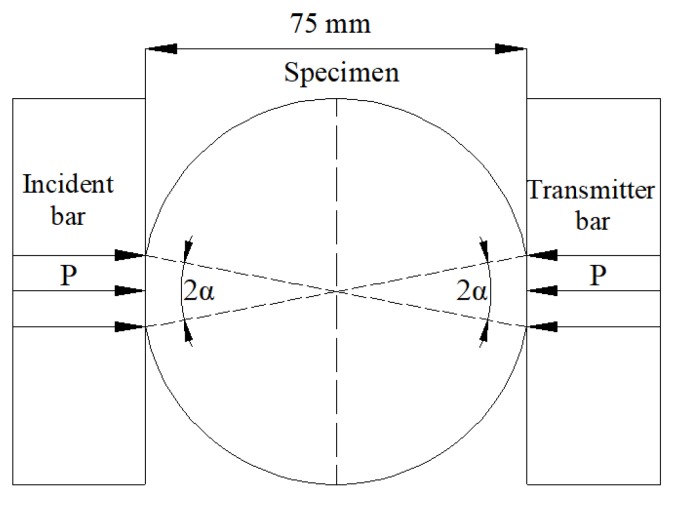
The flattened BD specimen in the SHPB test.

Figure 3 presents the BD-shaped specimen under radial dynamic loading. During SHPB tests of BD specimens, the cracks may initiate from the center and then propagate in a radial direction. Wang et al. [42] put forward that if specimens’ two planes are parallel to the elastic bars’ planes, and the degree of smoothness not less than 0.05 mm. The loading areas corresponding to the center angle 2α to meet 20° ≤ 2α ≤ 30°. Then the fracture behaviors can initiate from the specimens’ center. Vaseline should be wiped on the contact areas between the specimens and the elastic bars before the specimens are tightened between the elastic bars. Forces and velocities at both sides of the specimens can be calculated by the following equations [43]:

(4) $$P_{\mathrm{input}}\left(t\right){=S}_{B}E\left(\varepsilon_{i}\left(t\right){+\varepsilon }_{r}\left(t\right)\right){,\;V}_{\mathrm{input}}\left(t\right){=C}_{0}\left(\varepsilon_{i}\left(t\right){-\varepsilon }_{r}\left(t\right)\right)$$

(5) $$P_{\mathrm{output}}\left(t\right){=S}_{B}{E\varepsilon }_{t}\left(t\right){,\;V}_{\mathrm{output}}\left(t\right){=C}_{0}\varepsilon_{t}\left(t\right)$$

where $P_{\mathrm{input}}{,\;P}_{\mathrm{output}}{,\;V}_{\mathrm{input}}{,\;V}_{\mathrm{output}}$ are the forces (kN) and particle velocities (km/s) at the interfaces. $S_{B}{,\;E,\;C}_{0}$ are the elastic bars’ cross-sectional area (mm^2^), Young’s modulus (GPa), and wave velocity (km/s). $\varepsilon_{i}\left(t\right){,\;\varepsilon }_{r}\left(t\right){,\;\varepsilon }_{t}\left(t\right)$ are the strain pulses in the specimens. 

### 2.5. Experimental Tests

In this experimental study, the specimens’ strain rates were controlled by changing the gas pressure of the SHPB device power system. Specimens were subjected to impact loadings under the gas pressure determined by 0.15 MPa, 0.2 MPa, 0.25 MPa, 0.3 MPa, and 0.35 MPa to obtain the dynamic tensile strength of the concrete containing different SF levels under a wide range of strain rates. Those gas pressures correspond to impact velocities of 5.88 m/s, 7.38 m/s, 9.26 m/s, 10.46 m/s, and 11.37 m/s.

## 3. Experimental Results

The experiments were conducted by an SHPB device with a pulse-shaping technique under five different impact velocities. Mechanical test results of the prepared BD concrete specimens are reported below and the experimental results are exhibited using the tables and figures in this section.

### 3.1. Failure Pattern

During the dynamic loading tests, it is essential to distinguish which test is valid. Three necessary conditions were summarized by Chen et al. details can be seen in [6].

**Figure 4:**
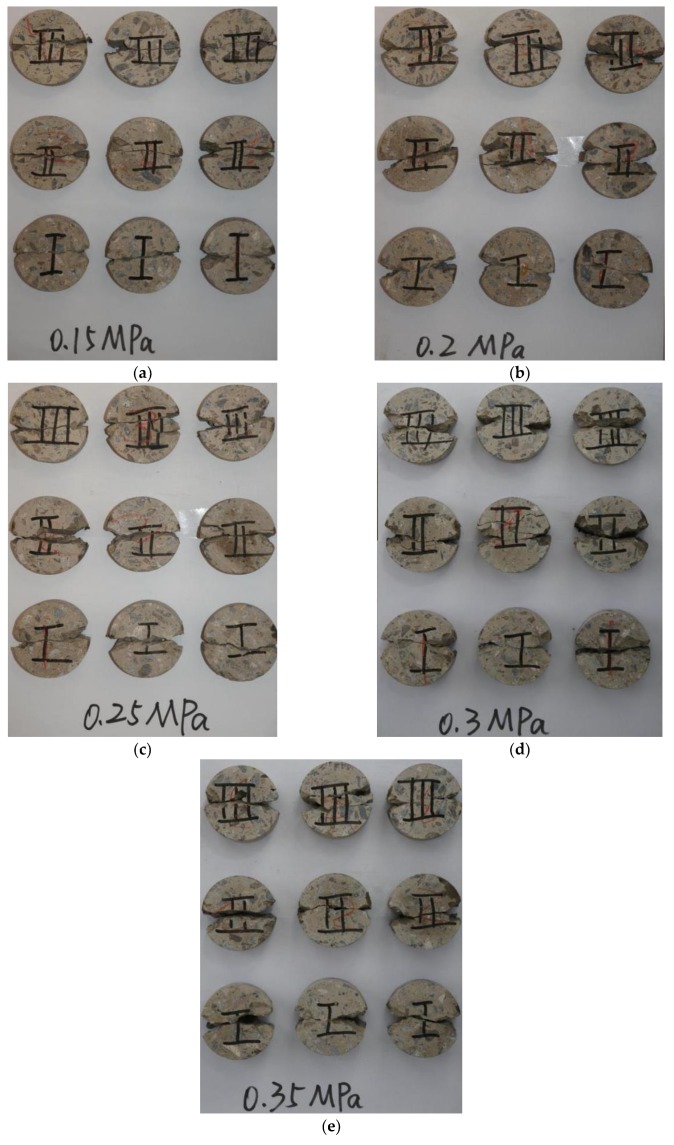
Failure patterns of three series of concrete specimens under different impact velocities: (**a**) *v* = 5.88 m/s; (**b**) *v* = 7.38 m/s; (**c**) *v* = 9.26 m/s; (**d**) *v* = 10.46 m/s; (**e**) *v* = 11.37 m/s.

**Figure 5:**
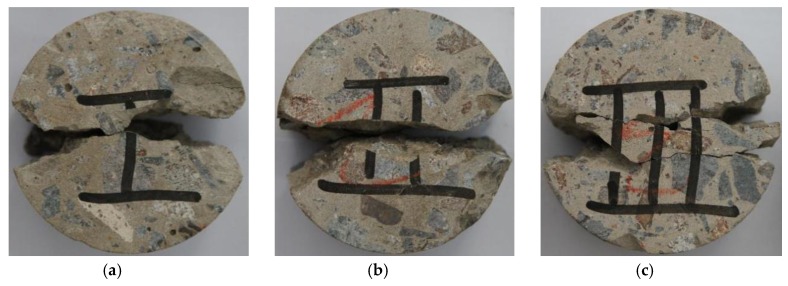
Schematics of substantial damage and missing edge details under impact velocity v = 11.37 m/s: (**a**) Concrete series I; (**b**) Concrete series II; (**c**) Concrete series III.

Based on the impact velocities, tests were classified into five groups: e.g., Group 1, the launcher pressure is 0.15 MPa, corresponding to velocity = 5.88 m/s. Figure 4 presents the typical failure patterns of BD specimens under dynamic splitting loading. Cracks started from the middle of the specimens and propagated along the loading radial direction to the platforms at both ends of the specimen. Finally, the specimens were fractured and damaged. There were also obvious fracturing phenomena near the platforms at both ends. The dynamic failure was violent and decisive, which resulted in tensile splitting along the loading axis, substantial damage, and missing edges (see details in Figure 5) of the broken halves at the loading areas. The broken edges of the specimens crushed into some small fragments at high strain rates. 

**Figure 6:**
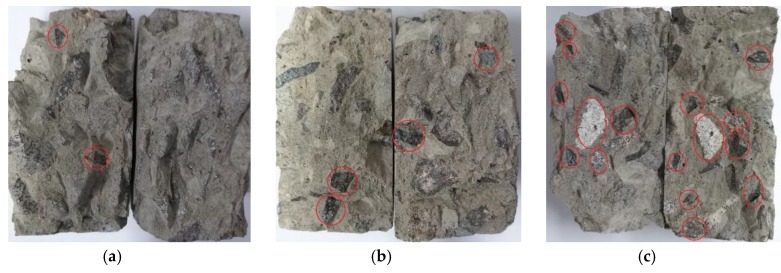
Typical fracture surfaces of concrete specimens under different strain rates: (**a**) Strain rate: $\dot{\varepsilon }$ = 45 s^−1^; (**b**) Strain rate: $\dot{\varepsilon }$ = 70 s^−1^; (**c**) Strain rate: $\dot{\varepsilon }$ = 105 s^−1^.

Figure 6 presents the typical fractured surfaces of concrete specimens under different strain rates. Usually, cracks just pass through mortar, and propagate along the interfaces between the mortar and aggregates under quasi-static loading. Under high strain rates, the stress increased so rapidly that cracks propagated through mortar, aggregates, and the interfaces between them. In addition, the number of aggregates that are fractured increases along the fractured surfaces with the increasing strain rate (see Figure 6a–c). The higher the loading strain rate, the more aggregates that are fractured.

Under the action of dynamic loading, failure will occur at a larger stress value compared with quasi-static loading. Table 6 presents the dynamic tensile strength and strain values at the maximum stress level. The peak stress was recorded as the dynamic tensile strength for each test. As can be seen from the table, there is a gradual increase in the dynamic tensile strength when more cement has been replaced with SF. This phenomenon can be explained by the physical properties of the micro-structure in concrete. This is because strength is directly related to the porous structure of concrete. The physical properties of SF which can fill cement particle gaps build up internal pressure. In other words, the dynamic tensile strength of concrete mixtures containing SF can be improved significantly when capillary porosity decreases. 

### 3.2. Stress–Strain Behavior

**Figure 7:**
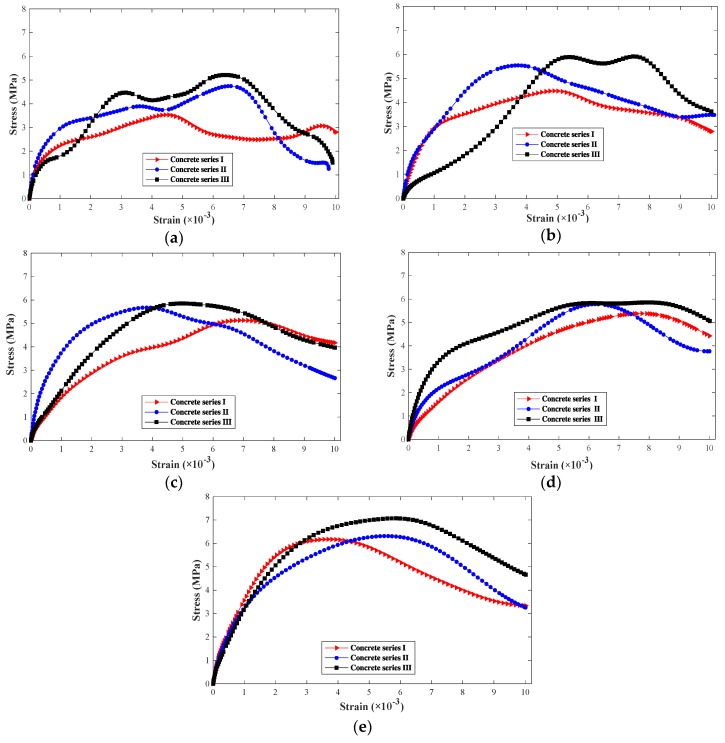
Dynamic stress-strain curves under different impact velocities: (**a**) 5.88 m/s; (**b**) 7.38 m/s; (**c**) 9.26 m/s; (**d**) 10.46 m/s; (**e**) 11.37 m/s.

Stress–strain behavior is one of the important characteristics for concrete-like materials, which can reflect their strength and deformation properties during loading processes [24]. In order to fully comprehend the dynamic response of concrete with different SF levels, Figure 7 shows the complete stress–strain behavior of concrete specimens under different SF levels and impact velocities. In the tests, the stress–strain rule trend of different specimens is consistent. Figure 7 presents the most representative group selected. It should be noted that the stress–strain behaviors of concrete are significantly sensitive to strain rates. At a given SF level, the peak stress and ultimate strain of the curve increase with the increase of loading velocities. High-speed loading can enhance the concrete strength. Moreover, for a given impact velocity, the peak stress generally increases with the increase of the SF level. Consequently, the results show that the strength of concrete is influenced by the loading rate and the SF level.

### 3.3. Strain Rate

The mechanical properties and dynamic behaviors of brittle materials relate to the strain rate [24]. Therefore, concrete is a typical strain rate-sensitive material. Concrete specimens may be fractured in the early stage of deformation. In this study, the peak stress–strain rate is taken as the representative strain rate of the dynamic tensile strength.

**Figure 8:**
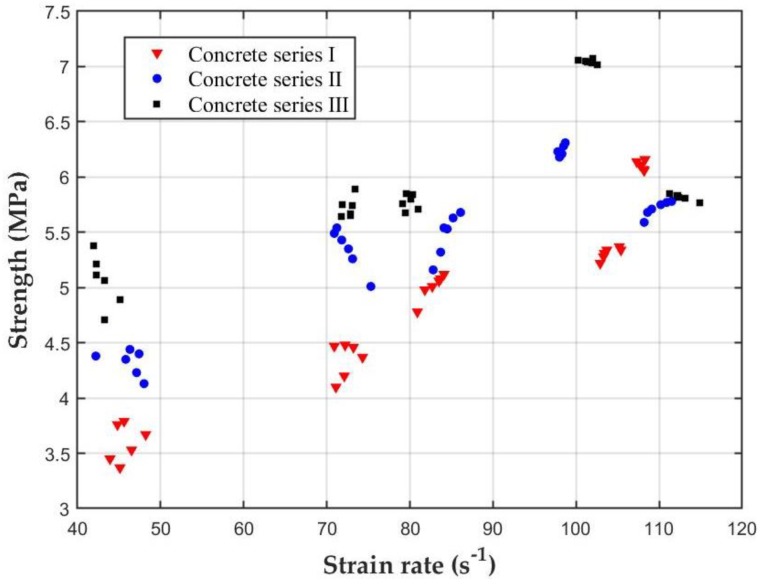
Relationship between the dynamic tensile strength and the strain rate of concrete with different SF rate levels.

Figure 8 presents the relationship between the dynamic tensile strength and the strain rate under different impact velocities. Based on the presented results, the concrete with SF is a kind of typical strain rate-sensitive material and presents higher strength at higher strain rates. Hence, the values of the dynamic tensile strength are higher than those under quasi-static loading. The results show that with the increase of the SF replacement, the dynamic tensile strength of each series of concrete increases with the increase of the strain rate. The dynamic tensile strength of each series of concrete increases with the increase of the strain rate and the SF replacement level. The tensile strength of concrete series III is higher than that of the other two series of concrete.

### 3.4. Dynamic Increase Factor (DIF)

The dynamic increase factor (DIF) can be defined as the dynamic strength divided by the quasi-static strength. Usually, the DIF can be employed to describe brittle materials’ sensitivity to the strain rate similarly to dynamic compressive tests [26]:

(6) $$\mathrm{DIF}=\frac{f_{\mathrm{td}}}{f_{\mathrm{ts}}}$$

where $f_{\mathrm{ts}}$ is the quasi-static tensile strength and $f_{\mathrm{td}}$ is the dynamic tensile strength. To investigate the behavior of the DIF in concrete series I, II, and III, different references provided the recommended empirical formulas via a logarithmic transformation of the strain rate [26]:

(7) $$DIF=A\cdot \log\dot{\varepsilon }-B$$

where $\dot{\varepsilon }$ is the strain rate ($s^{-1}$). The estimated results for different concrete by the least-square fitting method is shown in Table 7. The maximum absolute error (Error 1) and the mean square error (Error 2) are calculated to compare the difference between experimental and fitting values.

**Figure 9:**
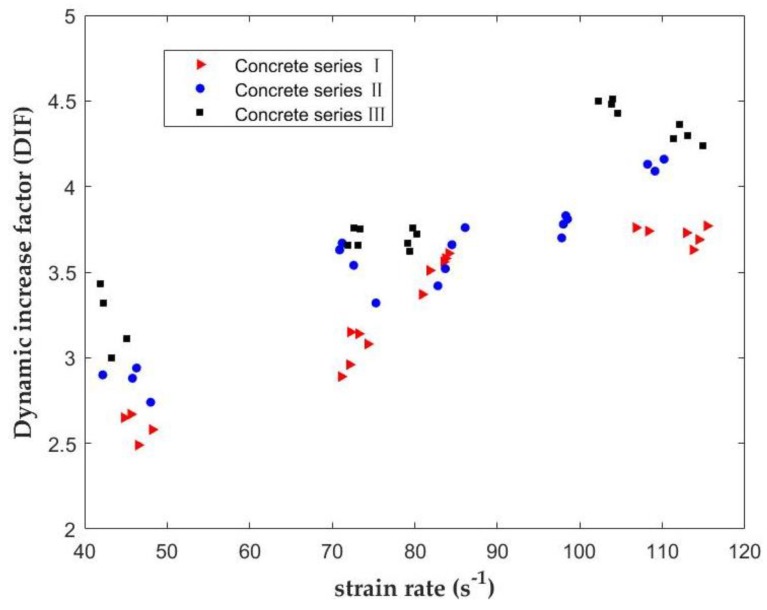
Relationship between the tensile DIF and the strain rate.

**Figure 10:**
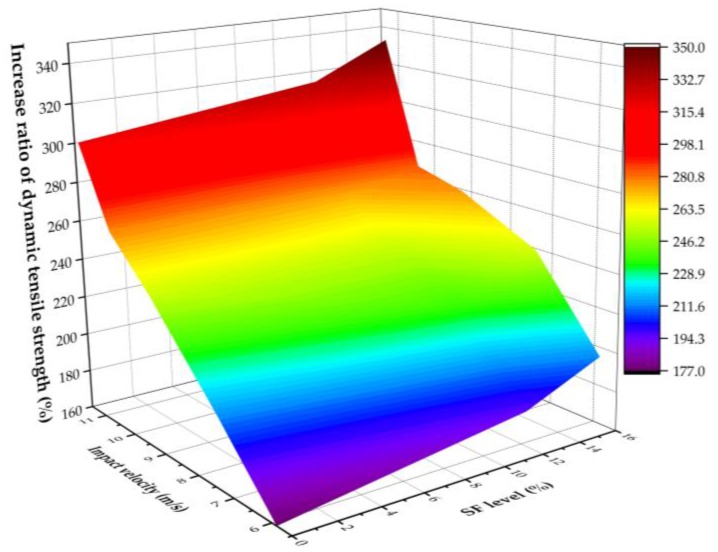
The increase ratio of dynamic tensile strength under different impact velocities and SF levels.

The relationship between the tensile DIF of three series of concrete and the strain rate is presented in Figure 9. The tensile DIF of three series of concrete increases with the increase of the strain rate. The dynamic tensile strength enhancement may be affected by the presence of crack growth and free water in the concrete [44]. Figure 10 presents the increase ratio of dynamic tensile strength under different loading velocities and SF levels. The increase ratio of the dynamic tensile strength in concrete containing 12% SF, for example, is 195%, 237%, 256%, 273%, and 332% at impact velocities of 5.88 m/s, 7.38 m/s, 9.26 m/s, 10.46 m/s, and 11.37 m/s, respectively.

### 3.5. Critical Strain

**Figure 11:**
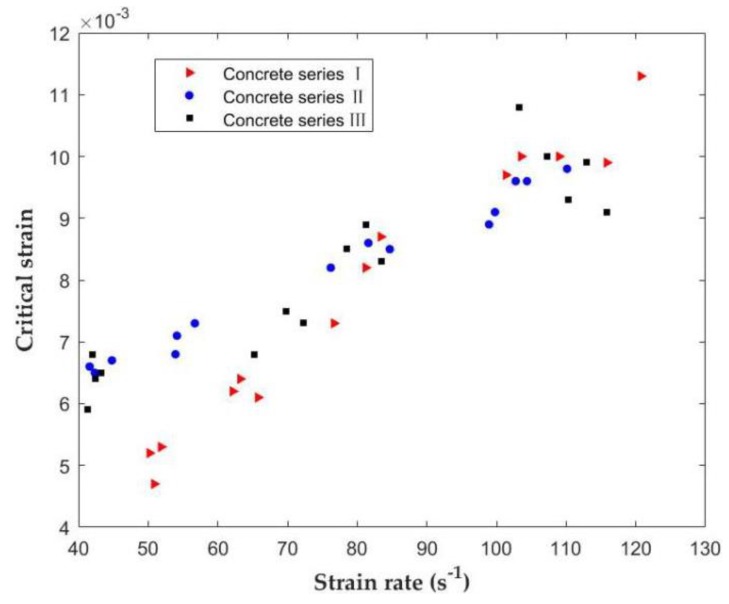
Relationship between the critical strain and the strain rate of concrete under different SF levels.

**Figure 12:**
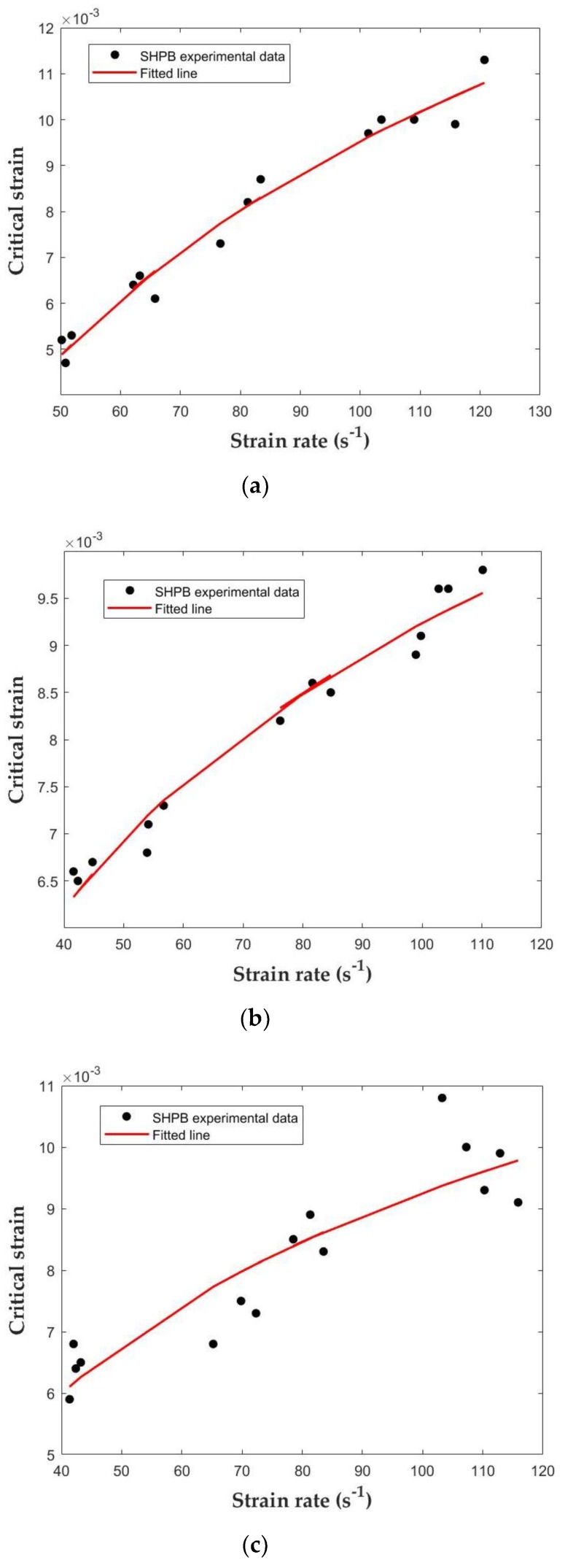
Fitted relationship between critical strain and strain rate: (**a**) Concrete series I; (**b**) Concrete series II; (**c**) Concrete series III.

The strain at the peak of stress is defined as the critical strain. Previous studies have shown that there are many disputes about whether the critical strain is related to the strain rate [45,46,47]. Watstein [45] revealed that the critical strain increases with the strain rate. On the contrary, Harris [46] pointed out that the critical strain decreases as the strain rate goes on. Harsh [47] even believed that the critical strain is not affected by the strain rate. Figure 11 presents the relationship between the critical strain and strain rate. It can be observed that, under impact load, the critical strain increases with the increase of the strain rate. Different from the tensile strength DIF, the increase of the critical strain of the three series of concrete at high strain rate is similar, which is not affected by the types of concrete. According to the influences of SF on the mechanical properties and dynamic behaviors of concrete under different loading velocities, the critical strain is directly related to the logarithmic function of strain rate in concrete series I, II, III, stated as [27]:

(8) $$\varepsilon_{cr}=A\cdot \log\dot{\varepsilon }-B$$

where,$\dot{\varepsilon }$ is the strain rate ($s^{-1}$). Table 8 indicates the estimated results for different concrete by the least-square fitting method. In order to better compare and study the difference between experimental and fitting values, the Error 1 and Error 2 are calculated. The corresponding fitting curves are depicted in Figure 12.

According to studies from Bischoff and Perry [48,49], with the increase of the strain rate, the degree of cracking required for failure increases, and the critical strain increases significantly under the action of impact loading. The increase in the critical strain can be explained by lateral constraints, which lead to the formation of many microcracks but prevent the formation of large macrocracks [50].

## 4. Conclusions

In this study, the effect of SF in concrete on the mechanical properties and dynamic behaviors of concrete under impact loading were investigated. With SF replacing cement, a series of changes have taken place in the physical structure and chemical composition of concrete. SF is particularly recommended as an alternative to moderate amounts of cement to obtain high-performance concrete with better mechanical properties. The SF in concrete gives better results and performance on mechanical properties under dynamic tensile loading. The dynamic tensile strength of specimens increases with the increase of the strain rate due to the excellent physical and mechanical properties of SF, and the stress-strain behaviors of concrete have a significant sensitivity to the strain rate. 

From the above results, it is observed that strain rate sensitivity is one of the important factors affecting the performance of concrete under impact loading. The failure mode of specimens will change with the increase of the strain rate. However, the strain rate sensitivity of the critical strain has little relationship with concrete series. In addition, the impact stress with respect to the cracking of concrete is a major issue under dynamic loading, but difficult to attain. Using more advanced instruments to obtain the impact stress, and proposing the most plausible explanation, are the important targets for the next research step.

## Figures and Tables

**Table 1:** Mix proportion of concrete with different SF levels by weight.

Mass of Concrete Ingredients (kg/m^3^)					
Items	Water	Cement	SF	Fine Aggregate	Aggregate
I	210.00	389.00	-	614.00	1141.00
II	210.00	340.80	48.20	614.00	1141.00
III	210.00	326.28	62.72	614.00	1141.00

**Table 2:** The chemical composition of OPC and SF.

Constituents	Chemical Composition (%)	
	OPC	SF
Loss on ignition	2.48	2.10
Silicon Dioxide	19.01	93.67
Calcium Oxide	66.89	0.31
Magnesium Oxide	0.81	0.84
Phosphate (P2O5)	0.08	-
Sodium Oxide	0.09	0.40
Potassium Oxide	1.17	1.10
Manganese Oxide	0.19	0.84
Aluminum Oxide	4.68	0.83
Ferric Oxide	3.20	1.30
Sulphur Trioxide	3.00	0.16

**Table 3:** The compressive strength and the tensile strength of tested concrete under quasi-static loading.

Concrete Series	Strength (MPa)	
	Compressive Strength	Tensile Strength
I	19.73	1.78
II	21.54	2.03
III	23.03	2.32

**Table 4:** Technical parameters and accuracy of the MTS testing machine.

Technical Parameters	Values
Maximum test force	10,000 N
Load measurement accuracy	≤±0.5%
Deformation measurement accuracy	≤±0.5%
Test table displacement measurement accuracy	≤±0.5%
Loading speed	10–500 mm/min
Speed Accuracy	≤±0.5%
Data sampling frequency	200 times/s

**Table 5:** Technical parameters and accuracy of the SHPB testing system.

Technical Parameters	Values
Pressure range	0.1–1.5 MPa
Strain rate range	10^2^–10^4^ s^−1^
Loading velocity	≤50 m/s
The diameter of elastic bars	74 mm
Speed Accuracy	≤±0.5%
Data sampling frequency	200 times/s

**Table 6:** Dynamic properties of three series of concrete specimens under different impact velocities.

Impact Velocity (m/s)	Concrete Series	Peak Stress (MPa)	Strain Rate at Peak Stress (s^−1^)
5.88	I	3.53	46.1
	II	4.75	43.2
	III	5.21	42.3
7.38	I	5.75	74.1
	II	5.90	68.8
	III	8.34	70.2
9.26	I	5.68	83.3
	II	5.85	86.1
	III	8.74	80.3
10.46	I	5.79	102.9
	II	5.86	106.7
	III	9.49	107.3
11.37	I	6.18	108.1
	II	9.45	106.8
	III	11.70	105.7

**Table 7:** The estimated results for different concrete using Equation (7).

DIF	A	B	Error 1	Error 2
Series I	1.3190	2.4693	0.2651	0.1308
Series II	1.2868	2.0404	0.2227	0.1254
Series III	1.2566	1.5996	0.3359	0.1744

**Table 8:** The estimated results for different concrete in Equation (8).

Critical Strain	A	B	Error 1	Error 2
Series I	0.0068	0.0219	6.2080 × 10^−4^	2.7308 × 10^−4^
Series II	0.0033	0.0060	3.8830 × 10^−4^	1.6671 × 10^−4^
Series III	0.0036	0.0072	0.0014	4.9670 × 10^−4^
